# Quali–Quantitative Characterization of Volatile and Non-Volatile Compounds in *Protium heptaphyllum* (Aubl.) Marchand Resin by GC–MS Validated Method, GC–FID and HPLC–HRMS^2^

**DOI:** 10.3390/molecules26051447

**Published:** 2021-03-07

**Authors:** Alberto Asteggiano, Andrea Occhipinti, Andrea Capuzzo, Enrica Mecarelli, Riccardo Aigotti, Claudio Medana

**Affiliations:** 1 Department of Molecular Biotechnology and Health Sciences, University of Torino, Via Pietro Giuria 5, 10125 Torino, Italy; enrica.mecarelli@unito.it (E.M.); riccardo.aigotti@unito.it (R.A.); claudio.medana@unito.it (C.M.); 2 Biosfered S.r.l. Via Paolo Veronese 202, 10148 Turin, Italy; 3 Abel Nutraceuticals S.r.l. Via Paolo Veronese 202, 10148 Turin, Italy; a.occhipinti@abelnutraceuticals.com (A.O.); a.capuzzo@abelnutraceuticals.com (A.C.)

**Keywords:** *Protium heptaphyllum*, Breu branco, oleum resin, amyrin, GC–MS, HPLC–APCI–HRMS, validated method, chemical profiling, triterpenes, volatile compounds

## Abstract

*Protium heptaphyllum* (Aubl.) Marchand (PH) trees are endemic to the tropical region of South America, mostly Brazil. Antibacterial, antinociceptive, anti-inflammatory, anxiolytic, antidepressant and anti-hyperlipidemic/anti-hypercholesterolemic effects were reported for its resinous exudate *Protium* *heptaphyllum* resin (PHR). This work aims to provide a qualitative and quantitative consistent chemical profiling of the major constituents of this resin and two extracts enriched in acid (acidic triterpene concentrated extract, ATCE) and neutral triterpenes (α and β-amyrin concentrated extract, AMCE). GC–MS/GC–FID was used for volatile terpene fraction, a validated GC–MS method was developed for quantification of neutral α and β-amyrin and HPLC–APCI HRMS^2^ was used for acidic triterpenes analysis. The chemical investigation reported 29 molecules, including 14 volatile terpenes, 6 neutral triterpenes and 11 acid triterpenes. The most abundant compounds were α-amyrin (251.28 g kg^−1^, 123.98 g kg^−1^ and 556.82 g kg^−1^ in PHR, ATCE and AMCE, respectively), β-amyrin (172.66 g kg^−1^, 95.39 g kg^−1^ and 385.58 g kg^−1^ in PHR, ATCE and AMCE, respectively), 3-oxo-tirucalla-7,24-dien-21-oic acid (80.64 g kg^−1^, 157.10 g kg^−1^ and 15.31 g kg^−1^ in PHR, ATCE and AMCE, respectively) and 3α-hydroxy-tirucalla-8,24-dien-21-oic acid (77.71 g kg^−1^, 130.40 g kg^−1^ and 11.64 g kg^−1^ in PHR, ATCE and AMCE, respectively). Results showed specific enrichment of acidic and neutral triterpenoids in the two respective extracts.

## 1. Introduction

The genus *Protium* is the most heterogeneous and abundant of the Burseraceae family, and the 135 known species that spread throughout the Amazon region, mostly in Brazil, make this genus the second most hyperdominant genus in the Amazon. Since ancient times, the oleum resin of *Protium heptaphyllum* (Aubl.) Marchand that is exudated from resiniferous ducts has been collected by the Amazon local population of Quilimbola. Oleum resin of *Protium heptaphyllum* is called by different popular names: Breu, Breu Branco, Olíbano Brasileiro, Resina de Almecéga, Almecegueira/Almesca Resin and Amazonic White Pitch [1,2,3,4]. The *P. heptaphyllum* oleum resin is a rich source of biologically active volatile and nonvolatile terpenes. Both these groups of secondary metabolites were recognized as critical factors in plant defense, since they can mediate the plant–herbivore interaction in both direct and indirect defenses through attraction/repelling of herbivores and the recruitment of herbivore predators/parasites, respectively [5].

The oleum resin is traditionally used by Amazon local population for the manufacturing of varnishes, wood boat sealants, fumigants, insect repellents and aromatic incense for religious rituals [1,2]. More recently, the antibacterial and antifungal potential of volatile terpenes in *P. heptaphyllum* oleum resin and leaf essential oils have been investigated against *Streptococcus mutans*, *Escherichia coli*, *Staphylococcus aureus*, *Serratia marcescens*, *Klebsiella pneumoniae*, *Proteus mirabilis* and *Candida albicans* [6,7].

Chemical investigations of nonvolatile fraction of *P. heptaphyllum* oleum resin have revealed the presence of α and β-amyrin, pentacyclic triterpenes that belong to the ursane and oleanane groups, respectively. They are the most abundant components of *P. heptaphyllum* resin (PHR). However, the nonvolatile fraction also includes a mixture of other triterpenes of oleanane, ursane and tirucallane series and, to less extent, lupane, taraxane and friedelane series [1,8]. Phytochemical investigations of *Protium heptaphyllum* have been usually conducted on oleum resin essential oils from different *Protium* species, mainly by GC–FID and GC–MS techniques [1,9,10]. In other studies, chemical derivatization and GC–MS analyses were performed in order to characterize and quantify α and β-amyrins and triterpenes in oleum resin extracts of *Protium* species by the use of toxic organic solvents, such as hexane, chloroform and dichloromethane [8,11,12]. Alternatively, ^1^H- and ^13^C-NMR were performed in order to investigate the triterpenes present in the nonvolatile fraction [13], though both NMR techniques suffer low feasibility due to high analytical costs and complex sample preparation.

Previous studies established anti-inflammatory, antinociceptive, antioxidant, gastroprotective and hepatoprotective effects of PHR and α and β-amyrins [14,15]. However, in order to exploit the *P. heptaphyllum* oleum resin and its extracts as natural remedies for the above reported biologic activities, it is important to carry out a complete chemical characterization and an accurate quantification of their constituents to be able to relate the observed biological effects with specific natural oleum resin components or classes. Therefore, the development of robust and validated analytical methods to characterize and quantify bioactive triterpenes in *P. heptaphyllum* is a key point to achieve the accurate phytochemical analysis and the standardization of food-grade oleum resin extracts, as well.

This study aimed to develop a validated quantitative method by mass spectrometry coupled with gas chromatography to accomplish an accurate quantification of bioactive triterpenes in PHR and two different PHR food-grade extracts: an acidic triterpene concentrated extract (ATCE) and an α and β-amyrin concentrated extract (AMCE). High pressure liquid chromatography coupled with high resolution mass spectrometry was used to achieve the phytochemical identification and quantification of acidic triterpenes in the PHR and its extracts. The development of a new HPLC–APCI HRMS^2^ method provided, for the first time, a reproducible analytical protocol to explore the complex mixture of triterpenes of this oleum resin that does not require chemical derivatization. A total of 29 molecules were identified and quantified in both oleum resin and food-grade extracts (ATCE and AMCE), reaching a comprehensive chemical characterization of the terpene fractions by a complete set of chromatography and mass spectrometry techniques.

## 2. Results

### 2.1. Volatile Terpenes Identification and Quantification

The most characterized constituents in *Protium* species are volatile compounds. Therefore, qualitative and quantitative analyses of volatile organic compounds (VOCs) in resin and both extracts were performed to assess *P. heptaphyllum* phytochemical markers. Results from gas chromatography analyses showed the presence of 14 VOCs. These compounds were identified by an analytical standard mix, NIST database and FFNSC3 Shimadzu Mass Spectra library (see Material and Methods) (Table 1). Volatile fraction in PHR and ATCE was characterized by the presence of several monoterpene hydrocarbons (α-thujene, α-pinene, sabinene, β-pinene, α-phellandrene, δ-3-carene, *p*-cymene; β-phellandrene; α-terpinolene) and oxygenated monoterpenes (1,8-cineole, *trans*-verbenol, terpinen-4-ol, *p*-cymen-8-ol), as well as the sesquiterpene β-*E*-caryophyllene. The most abundant VOCs in PHR were α-pinene 1.26% *w*/*w*, *p*-cymene 1.11% *w*/*w*, δ-3-carene 0.70% *w*/*w*, β-phellandrene 0.67% *w*/*w* and α-phellandrene 0.42% *w*/*w*. The total content of VOCs in PHR amounts to 5.07% *w*/*w*.

The samples ATCE and AMCE (crude food-grade extracts of PHR) showed quantitative changes in the percentage composition. In AMCE, *trans-*verbenol and *p*-cymen-8-ol were not detected; α-pinene, *p*-cymene, 1,8-cyneole, terpine-4-ol and β-*E*-caryophyllene were reduced, whereas sabinene, α-phellandrene, δ-3-carene, β-phellandrene and terpinolene showed higher concentration with respect to PHR. In ATCE, α-pinene, sabinene were reduced, while low volatile compounds (δ-3-carene, 1,8-cyneole, terpinolene, terpine-4-ol, *p*-cymen-8-ol and β-*E*-caryophyllene) had higher concentration with respect to both PHR and AMCE. In particular, β-*E*-caryophyllene in ATCE reached the 11.23% among the total VOCs, with respect to PHR (4.25%).

### 2.2. Method Validation for Amyrins Quantification

Instrumental quantitation of neutral triterpenes in the oleum resin of *P. heptaphyllum* and the two extracts (ATCE and AMCE) was performed upon diethyl ether solvent extraction and by gas chromatography coupled to mass spectrometry.

**Figure 1:**
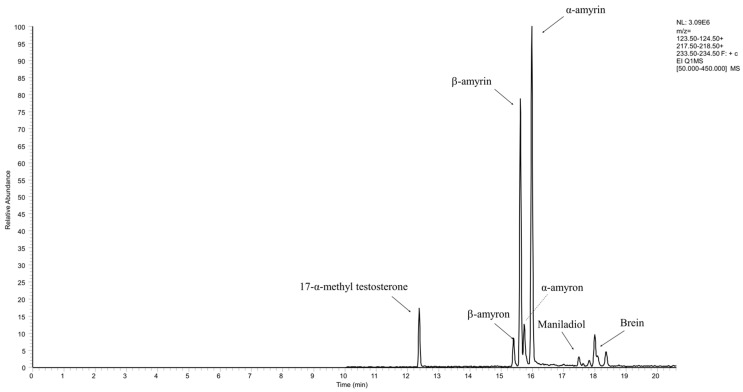
The figure shows the separation in PHR of 17-α-methyl testosterone (internal standard) (124 *m*/*z*), β-amyron (218 *m*/*z*), β-amyrin (218 *m*/*z*), α-amyron (218 *m*/*z*), α-amyrin (218 *m*/*z*), maniladiol (234 *m*/*z*) and brein (234 *m*/*z*) by their extracted ion chromatogram (EIC), as reported in brackets.

The separation of the two isomers, α- and β-amyrin, occurs with complete resolution in the isothermal zone at 320 °C (Figure 1). The two isomers belong to nonacidic ursane and oleanane triterpenes groups, respectively. Therefore, both isomers show similar fragmentation spectra with 218 *m*/*z* as base peak and 203 and 189 *m*/*z* as main fragment ions. The only difference between α and β-amyrin spectra resides in 203 *m*/*z* peak abundance, which is slightly superior in β-amyrin (see supplementary materials, Figure S2).

Quantitation method for α and β-amyrins was validated according to FDA and EMA guidelines [17,18]. The concentration range of validation was between 0.1 to 10 mg L^−1^. Validation parameters investigated were linearity (DIFF%), selectivity (SEL%), sensitivity (limit of detection (LOD), limit of quantitation (LOQ)), percent standard deviation (RSD%) and precision (ERR%). Eight-point calibration curves were analyzed (0.1, 0.2, 0.5, 1, 2, 6, 8, 10 mg L^−1^), and three replicates performed in three different days were compared to ensure the best repeatability, selectivity and precision of the analytical method (Table 2) (formulas are reported in supplementary materials, Figure S1).

The recovery was observed to be quantitative for all the molecules: results were 85 ±13%, 101 ± 3% and 98 ± 1%, respectively, in PHR, ATCE and AMCE extracts. In Table S1 (supplementary materials), calculation errors of three calibration curves are reported according to EMA validation parameters [18]. Validation requires that the back calculated concentrations of the calibration standards should be within ± 15% of the nominal value, except for the LLOQ, which should be within ± 20% and at least 75% of the calibration, with a minimum of six calibration standard levels; recalculation errors are reported in Table S1. The reported data showed that the ERR% fit into the acceptability range only above 0.5 mg mL^−1^. To obtain an acceptable error at the lowest calibration points, a weighted regression system model (1/Y) was used, increasing the sensitivity of method (Table 2). In supplementary Table S2, the recalculation errors ERR% are reported also in the case of 1/Y weighted calibration.

### 2.3. Quantification of Neutral Triterpens

The developed and validated GC–MS method was used to quantify α- and β-amyrins and the occurring lipophilic triterpenoids in raw material, ATCE and AMCE extract samples. The quantitative data were summarized in Table 3. The presence of amyrones, the oxidized forms of amyrins, in position 3 due the occurrence of carbonyl group (C3=O) instead of hydroxyl (3-OH), previously reported by [11,13], was confirmed by NIST identification and, furthermore, by similar fragmentation pathway and molecular peaks decreased by two mass to charge units (424 instead of 426 *m*/*z*) with respect to amyrins. Additionally, two further triterpenes were found: brein (3b,16b-dihydroxy-olean-12-ene) and maniladiol. Their quantification was obtained with α-amyrin calibration curve for brein (ion 234 *m*/*z*) (oleanolic family) and β-amyrin calibration curve for maniladiol (ion 234 *m*/*z*) (ursanic family). In Figure S3, all found neutral triterpenes structures are proposed.

Molecules identified and quantified belong to the ursanic (α-amyrin, α-amyron and Brein) and oleanic (β-amyrin, β-amyron and Maniladiol) triterpenes classes; their presence has been reported in all the samples except for the amyrones in AMCE extract. Neutral triterpenes made up the 48% (*w*/*w*) of PHR, the starting raw material, with predominance of α and β-amyrin, 25.1 and 17.2% *w*/*w*, respectively; α and β amyron registered concentrations of, respectively 1.84 and 1.43% (*w*/*w*), while brein and maniladiol were 1.49 and 1.22% (*w*/*w*), respectively. The extraction process for the production of AMCE and ATCE lowered the total concentration of neutral triterpenes in ATCE to 27% (*w*/*w*), which lead to their enrichment in AMCE up to 98% (*w*/*w*). In both crude extracts, the most abundant neutral triterpenes were α and β amyrins. However, in AMCE α and β amyrins reached the 55 and 38% (*w*/*w*), respectively, whereas their concentration dropped to 12 and 9.5% (*w*/*w*) in ATCE extract. The oxidated amyrins, α and β amyrons, were found only in ATCE in concentration of 2.71 and 1.27% (*w*/*w*), while maniladiol and brein were the least abundant molecules among the three samples, respectively 1.6 and 2.1% *w*/*w* in AMCE and 0.94 and 1.28% in ATCE.

### 2.4. Identification and Quantification of Acidic Triterpenes

**Figure 2:**
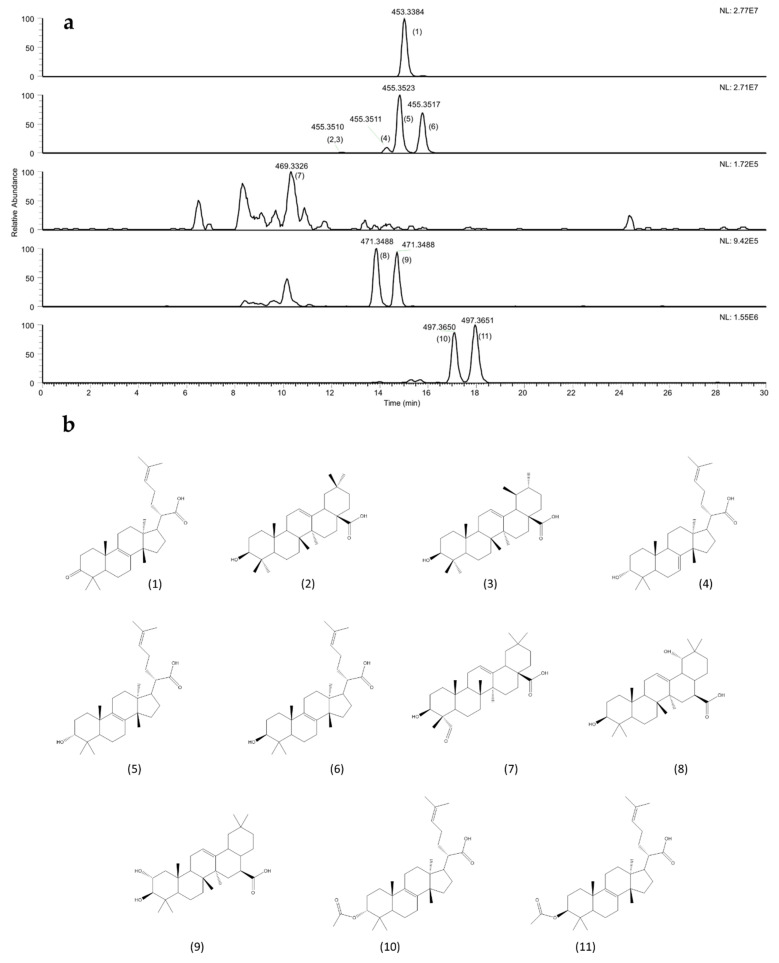
(**a**) Chromatographic separation of acidic triterpenes in ATCE sample; for each peak, *m*/*z* accurate value is reported. (**b**) Structure of proposed triterpenes: (1) 3-oxo -tirucalla-8,24-dien-21-oic acid, (2) oleanolic acid (3) ursolic acid (4) 3α-Hydroxy-tirucalla-7,24-dien-21-oic acid (5) 3α-Hydroxy-tirucalla-8,24-dien-21-oic acid; (6) 3β-Hydroxy-tirucalla-8,24-dien-21-oic acid; (7) gypsogenin; (8) siaresinol; (9) maslinic acid; (10) 3α Acetyl -tirucalla-8,24-dien-21-oic acid; (11) 3β-Acetyl-tirucalla-8,24-dien-21-oic acid.

An HPLC–APCI HRMS^2^ method was tailor-created to annotate the most abundant features among the acidic triterpenes. Molecules were identified in the instrumental raw data by their accurate mass-to-charge ratio, by their typical fragmentation pattern and using the specific analytical standards, when available. A total of 11 molecules were identified: 6 of them are tetracyclic triterpenes, whereas 5 are pentacyclic triterpenes. Identification was performed using specific analytical standards for oleanolic, ursolic and 3α-hydroxy-tirucalla-8,24-dien-21-oic acid (elemolic acid), while putative identification by exact mass and analysis of fragmentation pattern was carried out for the other triterpenes. In case of putatively annotated molecules, annotation level is the second, according to MSI [19]. Table 4 shows the identified or annotated compounds and their quantification, while Figure 2 shows the HPLC–HRMS chromatogram and molecular structures.

Qualitative analyses show that, in the samples, the most abundant compounds were always 3-oxo-tirucalla-8,24-dien-21-oic acid, 3α-hydroxy-tirucalla-8,24-dien-21-oic acid and 3β-hydroxy-tirucalla-8,24-dien-21-oic acid (respectively, elemonic acid, α- and β-elemolic acid). However, the extraction process affected the detection of less abundant compounds in AMCE: gypsogenin, siaresinol, 3α-acetyl-tirucalla-8,24-dien-21-oic acid and 3β-acetyl-tirucalla-8,24-dien-21-oic acid were not detected in this sample. Quantitation data were obtained by using moronic acid as analytical standard to build an external calibration curve. This acid triterpene, from the oleanane group, was selected due to its similarity with the identified molecules in the samples, for the high purity grade of the available standard and for its absence in PHR matrices, making it possible to also use it as an internal standard. Qualitative and quantitative differences were reported for the assayed samples. The total content of acid triterpenes was 23.4% *w*/*w* in the raw material, the *P. heptaphyllum* oleum resin. However, the extraction process led to an enrichment of acidic triterpenes in the ATCE sample, since their total content reached 40.6% *w*/*w*, whereas, in AMCE, we observed a drastic decrease of their content (up to 3.0% *w*/*w*).

## 3. Discussion

### 3.1. VOC Qualitative and Quantitative Characterization

*P. heptaphyllum* oleum resin is rich in volatile terpenes, mainly monoterpenes. The chemical profiling of VOCs in oleum resin samples from different geographical origins [2] and aging stages [20] are available in literature studies. Therefore, the VOCs profiles are intended to be the most rugged chemical parameter to carry out taxonomical evaluation of plant material. However, VOC fraction exhibits a chemical composition that can be strongly altered with time; in particular, the increase of oxidated monoterpenes was observed [14]. In the present study, the sample of oleum resin contains (limited to the VOC fraction) the 95.7% of monoterpenes with predominance of *p*-cymene (21.85%), δ-3-carene (13.90%) and β-phellandrene (13.33%), whereas β-(*E*)-caryophyllene was the only sesquiterpene quantified.

To our knowledge, this is the first report showing the qualitative and quantitative analyses of VOCs from PHR by using solvent extraction, while all previous investigations showed the qualitative percentage of volatiles in PHR essential oil. However, the present data on VOC content in PHR oleum resin confirm the previously published reports, regarding which monoterpenes are the main chemical constituents of volatile fraction in the range between the 85% and the 98% [9,21,22]. The higher percentage of monoterpenes in the analyzed PHR samples were α-pinene (24.85%), *p*-cymene (21.85%), 3-carene (13.90%), β-phellandrene (13.33%) and α-phellandrene (8.30%). In previously published studies, *p*-cymene and β-phellandrene were reported as the main volatile constituents of *P. heptaphyllum* essential oil (13.63% and 60.68% respectively) [21], whereas α-pinene and α-phellandrene were reported among the most abundant VOCs in essential oil reaching the 10.5% and the 16.70%, respectively, with terpinolene (28.50%) and limonene (16.9%) as main compounds [9]. The compound 3-carene was also reported among the characteristic *P. heptaphyllum* monoterpenes in essential oil (4.00%) from mechanically wounded tree [23] and commercial resin (5.11%) [22]. Few sesquiterpenes were reported in literature on *P. heptaphyllum* essential oil: β-(*E*)-caryophyllene was reported in two previously published reports at the percentage of 1.5% and 1%, respectively [9,11], whereas other notable reported sesquiterpenes were α-ylangene at 0.47% [21,23], α-cubebene (3.30%) [11] and α-gurjunene (0.30%) [9]. Our data confirms the limited presence of sesquiterpenes in PHR as it is reported in literature in essential oil analyses.

The reported composition of volatile fraction matches *P. heptaphyllum* profile, although differences in chemical composition and abundance of VOC profile may be related to different factors, such as method of extraction (solvent extraction vs. essential oil), seasonal variations, plant collection site and environmental conditions (phenotypic plasticity) and collection strategy from naturally exudates or tapped trunks [2].

### 3.2. Triterpenes Identification and Quantitation

Neutral triterpenes were analyzed by GC–MS technique, which takes advantage of the electronic ionization for the detection and quantification of this poorly ionizable class of molecules, whereas the acidic triterpene molecules in the samples were investigated by HPLC coupled to APCI HRMS^2^. The APCI ionization source is capable of ensuring a higher sensitivity for lower volatile, poorly chargeable and highly lipophilic molecules with single acidic moieties [24].

#### 3.2.1. Method Validation for Amyrin Quantification

The reported data show a validated quantitative method for α and β-amyrin that was developed according to FDA and EMA guidelines [18]. The gas chromatography validated method that we reported for the analysis of oleum resin and its extracts was fine-tuned in the concentration range with which they occur naturally to ensure a selective, repeatable and accurate quantification of these neutral bioactive triterpenoids. The proposed method can reach LOQ values ranging from 670 ppb for α-amyrin and 790 ppb for its β isomer up to 390 ppb and 350 ppb, respectively, using the weighted calibration. The use of a weighted calibration method reduced both LOD and LOQ by decreasing the ERR% parameter at 0.1 mg L^−1^, thus enhancing the sensitivity of the quantitative method.

In literature data, only one validated quantitative method for α-and β-amyrin was reported for quantitation of amyrins in rat plasma [25]. In the reported case, the method was developed to achieve great sensitivity and selectivity using a SIM (Selected Ion Monitoring) scan and allowed for reaching very low LOQ and LOD values in the concentration order of 0.001 mg L^−1^–1 ppb, as required by clinical analysis approaches that involve complex sample preparation, including purification and reconcentration steps.

Several literature studies report triterpenes quantitation methods in different food and botanical samples with similar validated quantitative approaches. In 2013, a GC–MS quantitative method for derivatized ursolic acid was reported in food supplement analysis with a LOQ of 10 mg kg^−1^ (10 ppm) [26]. Another work reports a HPLC–MS/MS validated method created for acidic triterpene analysis in botanicals and proposes LOQ values of 175 ng mL^−1^ (175 ppb), 108 ng mL^−1^ (108 ppb) and 85 ng mL^−1^ (85 ppb) for oleanolic acid, ursolic acid and betulinic acid, respectively. The lower quantitation limits in this latter case are related mostly to the higher sensitivity of the HPLC tandem mass spectrometry method proposed, made possible by the acidic nature of the analyzed molecules. A further study reports the validated method for the quantitation of triterpenes and sterols in the aerial part of *Justicia anselliana*. The method was validated for the lupeol triterpene; the authors chose to use GC–MS instrumentation for the identification and a GC–FID for the quantitation. The calibration curve was obtained with a weighted 1/X regression, and the method provided a LOQ of 5 mg L^−1^ (5 ppm). [27]. Therefore, the method proposed in this work has lower LOQ to the ones already reported with gas–chromatographic techniques, establishing a reliable and accurate quantitative method for the investigated matrices. Therefore, this is the first validated method for the quality control analysis of these specific phytochemical markers in plant raw material and extracts that can be easily adopted by dietary supplement industries.

#### 3.2.2. Neutral Triterpenes Quantitation

Amyrins and amyrones isomers, brein and maniladiol profiling in PHR has been thoroughly investigated in several publications, mostly by GC–FID [8,11]. However, there are no literature data proposing a complete quantitation approach with a MS detector. The α and β-amyrin quantitation results in PHR are in line with previously published data obtained by Silva et al. [11], which compared seven *Protium* species by GC–FID. In *P. heptaphyllum*, the authors reported a concentration of 40.9% among α, β-amyrin and α-amyrone. Unfortunately, the authors did not record β-amyrone, brein or maniladiol, and limited information was reported regarding the quantification method that could allow an accurate comparison with our data. Other literature data have shown quantitative differences with respect to our data [8]. In detail, Siani et al. observed α-amyrin concentrations ranging from 30 to 43%; this difference may be linked to the different quantitative approaches that the authors used. Siani et al. reported the use of the relative peak area by a GC–FID, while our work employs a specific calibration curve performed on α- and β-amyrin. Due to the relevant differences in the quantitative methods that were reported in literature, the abundance ratio between ursanic and oleanic triterpenes could be used to compare our results with data obtained by previous studies. In our work, the ursanic and oleanic triterpenes ratio is 1.3 in all analyzed samples (PHR, ATCE and AMCE). The observed value is slightly higher than Silva et al. (1.1) [11] and lower than Siani et al. (3.0) [8] and Neves et al. (2.0) [12]. Different ursanic–oleanic abundance ratios found by Neves et al. in their GC–MS quantitation experiment could be also explained by using α-amyrin as a unique calibration standard also applied to β-amyrin quantitation. In fact, data in our present work show that α-amyrin MS response factor (218 *m*/*z* quantifier ion) is substantially higher (1.2-fold) than its oleanic isomer. The observed difference in response factor could be responsible for β-amyrin underestimation and change in ursanic and oleanic triterpenes ratio if titrated by α-amyrin equivalents. Furthermore, data comparison confirms that significant natural variability between PHR samples can occur also in α- and β-amyrin content related to different factors, such as seasonal variations, plant collection site and environmental conditions (phenotypic plasticity) [28]. Therefore, the reported data highlight the importance of using a validated quantitative method to perform accurate comparisons and the relevance of the described GC–MS approach.

#### 3.2.3. Acidic Triterpenic Fraction Identification and Quantification

The proprietary extraction process for ATCE and AMCE production allows for the concentrate of acidic triterpenes in ATCE extract and the amyrins and other neutral oleanic and ursanic triterpenes in AMCE, respectively. The obtained data are the first quantitative report of the acidic triterpenes in *P. heptaphyllum* oleum resin and its derivates using a HPLC–APCI HRMS mass spectrometry approach. The use of a HPLC-based technique has a great advantage for this class of molecules, since does not need any derivatization or sample pretreatment. In contrast, gas–chromatographic approaches for the analysis of more polar analytes or ionized groups, such as carboxyl, need derivatization steps by formation of methyl esters or trimethyl siloxanes in order to lower their boiling point and reduce possible interactions with stationary phase [8,28].

We were able to identify four acidic triterpenes by using their analytical reference standards: 3-oxo -tirucalla-8,24-dien-21-oic acid (elemonic acid), oleanolic acid, ursolic acid (peak #1,2,3, Figure 2a and 3α-hydroxy-tirucalla-8,24-dien-21-oic acid (α-elemolic acid), (peak #5, Figure 2a). The presence of oleanolic and ursolic acids is also supported by their strong similarity with α- and β-amyrins, respectively, an ursanic and an oleanic triterpene. To further support the presence of oleanolic acid and ursolic acid in the PHR, it should be observed that α and β-amyrin are reported to be direct precursors of ursolic acid and oleanolic acid, respectively [29].

Interestingly, the peaks #4 and #6 (Figure 2a) show similar fragmentation pattern with respect to peak #5. We attempted a fragmentation-led recognition also referring to elder publication data [8]. In their paper, Siani et al. reported the presence of 3α-hydroxy-tirucalla-7,24-dien-21-oic acid, which is a B ring isomer of 3α-hydroxy-tirucalla-8,24-dien-21-oic acid with the double bond shifted from position C8 to position C7, and an epimer of 3α-hydroxy-tirucalla-8,24-dien-21-oic acid. Those molecules were also elucidated by crystal structure analysis [30,31]. The main difference in the spectral fragmentation of peak #4 with respect to peaks #5 and #6 is the presence of 339 *m*/*z* ion instead of 441 *m/z.* This 2 Dalton shift can suggest the different ring rearrangement due to a different position of the double bond. In particular, we assumed that the presence of the double bond in position 7 was more likely to form an aromatic rearrangement in ring B upon fragmentation, while the double bond in position 8 formed a less stable fragment with a nonaromatic ring. To further confirm this hypothesis, we found that 339 *m*/*z* ion in peak #4 had a higher abundance than 441 *m*/*z* ion in peaks #5 and #6. Finally, peak #6 showed the same fragmentation pattern of peak #5 and could be attributed to its C-3 epimer. In addition, 3-oxo -tirucalla-8,24-dien-21-oic acid, a tirucalladienoic acid oxidized derivative was identified by the use of its analytical standard and mass fragmentation interpretation. With respect to the work of Siani et al. [8], we found different relative abundances among these molecules. This can be related to 0the plasticity of metabolic profiles, which is typical in plants.

Fragmentation studies also allowed for the putative identification of two acetylate derivates of alpha and beta tirucalladienoic acids (peaks #10 and #11, Figure 2a), respectively 3α-acetyl-tirucalla-8,24-dien-21-oic acid and 3β-acetyl-tirucalla-7,24-dien-21-oic acid. Their putative identification was based on the previous assessment of the presence of two tirucalladienoic acid hydroxy-epimers (peaks #5 and #6, Figure 2a) and their identification in previous studies on *Protium* [8]. For the identification of other peaks, maslinic acid, gypsogenin and siaresinol, online software, such as Metfrag [32] and Metlin [33] databases, was used.

### 3.3. Reported Terpenes Biologic Activities

*p*-Cymene is the major constituent in VOC fraction in both PHR and ATCE. It is found in more than 100 plants belong to *Lamiaceae*, *Mirtaceae*, *Burseraceae* (such as *Protium*) and *Asteraceae* genus. This secondary metabolite has relevant antioxidant, anti-inflammatory, antinociceptive, anxiolytic and antimicrobial activities [34,35]. Β-Phellandrene, 3-carene and α-pinene have reported antibacterial and antifungal activities by disrupting the pathogen membrane structure and leading to energy metabolism dysfunctions in pathogen microorganisms [36,37,38]. β-(*E*)-caryophyllene is naturally present in several plant species and marketed as extracts due to its relevant commercial value. β-(*E*)-caryophyllene is defined as a dietary cannabinoid, due to its selective agonist activity against CB_2_ receptor on the CP55,940 binding site. Agonist ligands of CB_2_ receptors have been shown to inhibit inflammation and edema formation, to exhibit analgesic effects and to play a protective role in hepatic ischemia–reperfusion injury [39,40].

Triterpenoids have found to carry several beneficial health effects in vitro and in vivo [41]. The α and β-amyrins from *P. heptaphyllum* oleum resin showed antinociception in mice, in which orofacial pain was induced by capsaicin and formalin subcutaneous injection [42,43] or visceral pain, which was induced by cyclophosphamide and mustard oil (colonic inflammation) [44]. Putative interaction between α and β-amyrins and GABA-A receptor was also investigated in vivo, leading to the hypothesis of antidepressant effects carried out by PHR oral administration [6]. Anti-inflammatory effects were investigated in gastric ulcer model by oral administration in mice of oleum resin. The PHR, at 200 and 400 mg kg^−1^ b.w., significantly attenuated the gastric damage induced by both ethanol and acidified ethanol [45]. In periodontitis-induced inflammation, α- and β-amyrins pretreatment at 5 mg kg^−1^ significant reduced TNF-alpha, the gingival myeloperoxidase and thiobarbituric acid-reactive substances, retarding acute inflammation in rat model [46]. Moreover, several publications reported the impact of PHR on regulation of lipidemic and cholesterolemic levels. PHR administration showed a body weight reduction in HFD (High Fat Diet)-treated mice with a sibutramine-comparable efficiency; in addition, ghrelin decrease and leptin increase (typical in HFD control mice) were successfully modulated [45,47].

Several acidic triterpenoids have been found to carry beneficial health effects in human and animal models, such as anti-inflammatory, anti-microbial and antiviral agents. In HIV-1 infected H9 lymphocytes, it was reported as an in vitro HIV-protease inhibition by maslinic acid [48]. Moreover, oleanolic, ursolic and tirucallic acids have been long-recognized to have anti-inflammatory and antihyperlipidemic properties in vivo test [49,50], as well as 3-oxo -tirucalla-8,24-dien-21-oic acid, which also reports anti-proliferative activities [51]. In a recent study, oleanolic and ursolic acid have also shown in silico putative activity against the main protease of the SARS-CoV-2 responsible for coronavirus disease (COVID-19), suggesting further investigations for their potential antiviral activity [52,53,54].

## 4. Materials and Methods

### 4.1. Samples of Protium Heptaphyllum

Samples that were used in this study are listed and described in Table 5. The samples of oleum resin and crude extracts were from *Protium heptaphyllum* (Aubl.) Marchand was provided by Abel Nutraceuticals (Turin, Italy). The oleum resin was collected in Brazil in April 2019. The samples of crude extracts were obtained by Abel Nutraceuticals (Turin, Italy) (Batches # P75-2-0 and P75-2-1 used under the commercial brand Hepamyr^®^) through a patent pending hydroalcoholic extraction process and supplied in a powder form.

### 4.2. Chemicals

HPLC grade diethyl ether (DEE), dichloromethane (DCM), ethanol (EtOH) anhydrous, methanol (MeOH), cyclo-hexane (HEX); 17-α-methyl testosterone (M-TEST) (≥97%); terpenes analytical standard mix (Mix Cannabis Terpenes A–B); and C_7_-C_30_ n-alkanes mix were purchased from Sigma–Aldrich, Merck (Darmstadt, Germany); α and β-amyrin standards (≥98.5%) (CAS: 638-95-9 and 559-70-6), and ursolic acid (CAS: 77-52-1) were purchased from Extrasynthése (France); moronic acid (MA) (≥96%) oleanolic acid hydrate (CAS: 508-02-1) were purchased from TCI-EUROPE (Belgium). α-β elemolic acid (CAS: 28282-27-1) and elemonic acid (CAS: 28282-25-9).

### 4.3. Extraction of VOC and Neutral Triterpenes

Neutral triterpenes and volatile organic compounds (VOC) extraction from PHR, ATCE and AMCE was carried out with DEE. This solvent was chosen to include the widest range of molecule polarity. An aliquot of 20 mg of powdered samples was spiked with the internal standard (M-TEST), according to Ching et al. [25], to obtain a final concentration of 3 mg L^−1^ of M-TEST in the analyzed solution. Extraction was carried out with 20 mL DEE. The solution was bath-sonicated for 20 min, and the undissolved particles were precipitated by centrifugation at 8000× *g* for 5 min. The solution was then diluted in DCM or HEX, 1:50 or 1:10 depending on the matrix (1:50 for oleum resin and extract, 1:10 for GC–FID quantitation of VOC fraction) before analysis.

### 4.4. Extraction of Acidic Triterpenes

Extraction of acidic triterpene from PHR, ATCE and AMCE was performed with EtOH as follows: 50 mg of powdered sample were extracted with 10 mL of EtOH, then the solution was bath-sonicated for 20 min and precipitated by centrifugation at 8000× *g* for 5 min and finally diluted with MeOH.

### 4.5. VOC Profiling and Quantitation

Volatile terpenes were analyzed with a GC–FID/MS instrument (GCMS-QP2010 SE-Shimadzu, Japan). The VOCs were firstly identified with the MS detector and then quantified with the FID. The analyses were carried out with the same chromatographic condition for GC–MS and GC–FID. Samples (1 µL) were injected in an injection port kept at 280 °C in split mode (GC–FID) (Split Ratio 1:5) and splitless mode (GC–MS). Split flow was restored after injection at 3 mL min^−1^. The instrument used helium as carrier gas (column flow 1 mL min^−1^) and was equipped with a capillary column: Restek RXI 5-MS (30 m × 0.25 mm × 25 µm film thickness). The thermal gradient was set as follows: 0′ 50 °C, 30′ 140 °C, 45′ 320 °C, 55′ 320 °C. Mass spectrometry analysis was performed with an EI ionization source set at 70 eV, source temp: 200 °C, quadrupole temp: 150 °C, and the detector scan ranged between 50 and 650 *m*/*z*. GC–MS and GC–FID data were handled and managed with LabSolution (Shimadzu, Japan). MS analytes identification was achieved by NIST17 and FFNSC3 Shimadzu Mass Spectra libraries comparison and also by comparison of calculated linear retention indexes (LRIc) obtained with a C_7_-C_30_ n-alkanes mix with literature LRI (LRIL) [16]. Retention times and LRI were then used to identify the peaks in GC–FID chromatograms for quantitation. Quantitation was performed by external calibration with a standard mixture of VOC terpenes (Mix Cannabis Terpenes A–B, Sigma–Aldrich, see chemicals). Terpenes in sample which were not present in the standard mix were quantified with the nearest-eluting molecules in the standard.

### 4.6. Neutral Triterpenes GC–MS Detection and Quantification

The quantification of neutral triterpenes was carried out by a GC–MS. The instrument was composed by a TRACE 1310 and a TSQ Quantum Ultra QqQ (Thermo Scientific). Analysis was carried out by a temperature gradient, and detection was made in full-mass mode (50–450 *m*/*z* scan range). The instrumental separation conditions were injection volume: 0.5 µL, injector: PTV, splitless mode, constant temperature (280 °C), split flow: 50 mL min^−1^, carrier gas: He, 1.2 mL min^−1^, capillary column: Agilent DB-1 (30 m × 0.53 mm × 5 µm); T ramp: 0′ 150 °C, 4′ 150 °C, 12.5′ 320 °C, 20′ 320 °C. Detection parameters are listed as follows: ion source: EI 70 eV, source temperature: 250 °C. GC–MS data were managed with Thermo Xcalibur 3.0 software (Thermo Scientific). For the quantification, extracted ions were 218, 203, 426, 189 *m*/*z*. Analytical response of extracted ions (218 *m*/*z* for quantitation, 203, 426, 189 *m*/*z* for confirmation) was corrected by dividing their value by the analytical response of M-TEST (ion 124 *m*/*z*) in order to avoid any systematic error of analytical sensitivity.

### 4.7. Quantitation Method Validation

Method validation was performed in order to assess at first its repeatability and overtime stability; parameters studied were overtime linearity and repeatability (DIFF%), selectivity (SEL%), sensitivity in terms of LOD (limit of detection) and LOQ (limit of quantitation), percent standard deviation (RSD%) and imprecision (ERR%). Recovery was calculated respectively to the recovery of internal standard added in sample. Calculation equations used for validation are reported in supplementary materials (Figure S1).

### 4.8. Acidic Triterpenic Fraction HPLC–HRMS^2^ Identification and Quantification

An untargeted accurate tandem-mass-based identification by HPLC–HRMS^2^ approach was developed to determine the components among the acidic triterpenes. Instrumental setup was made out from a HPLC Dionex ultimate 3000 HPLC, coupled via an APCI as ion source with a LTQ-Orbitrap (Thermo Scientific). Separation was carried out with a Luna C18(2) (150 × 2 mm, 100 Å, 3µm-Phenomenex), 0.1% formic acid in water (solvent A) and acetonitrile (solvent B) as mobile phases. Solvent ramp was from 5 to 100% solvent B in 30 min, followed by a column reconditioning of 15 min. The solvent flow was set to 0.2 mL min^−1^, and injection volume was 10 µL. Detection parameters were the following: negative ion mode, capillary temperature: 250 °C, APCI Vaporizer Temp: 450 °C, sheath gas 35 Arb, auxiliary gas: 15 Arb, discharge needle: 5 kV; acquisition was carried out in dependent scan mode with mass range from 220 to 1000 *m*/*z*. Raw data were analyzed as follows: sequentially raw datafile were converted in mzXML files by MsConvert software (Proteowizard) [55] and submitted for deconvolution and annotation to XCMS online [56] software with following parameters: *Feature detection*- Method: centWave; ppm: 10; minimum peak width:10, max:120; mzdiff: 0.05; S/N threshold: 6; integration method: 1; prefilter peaks: 3; prefilter intensity: 100,000; noise filter: 0. *Retention Time Correction*- profStep:1; Alignment: minfrac:0.5; mzwid: 0.015; bw: 5; minsamp:1; max: 100. *Statistics*- Statistical test: Welch t-test; perform post hoc analysis: True; p-value threshold: 0.05; fold change threshold: 1.5; p-value threshold (significant): 0.005. *Annotation*- Search for: isotopes; *m*/*z* err: 0.05; ppm: 10. HPLC–HRMS data were managed with Thermo Xcalibur 3.0 software (Thermo Scientific). Therefore, to obtain the quantitation of identified acidic triterpenes in the samples, moronic acid was used as external standard to build calibration curve ranging from 0.1 to 15 mg L^−1^.

## 5. Conclusions

The numerous reports describing and demonstrating the biological activities of triterpenes from *Protium heptaphyllum* and other plant sources are driving a growing interest in exploiting the biologic potential of triterpene in this botanical source. However, natural chemical variability of oleum resin, that can be correlated to seasonal variations, plant collection site and environmental conditions, impairs the investigation and comparison of in vitro and in vivo mechanisms of action and the correlation dose/effects, as well as assessments of safety and bioavailability.

A combined analytical approach based on different chromatographic techniques was proposed to face the issue of chemical identification and quantification, as well as standardization in *P. heptaphyllum* oleum resin extracts. GC with FID or MS detectors was used for the identification and the quantification of VOCs and neutral triterpenes. A faster and more reliable solution has been developed and validated for bioactive neutral triterpenes in *P. heptaphyllum* oleum resin and two fractionated extracts enriched in acid (ATCE) and neutral triterpenes (AMCE) that can be of valuable interest for food supplements applications. Moreover, for the first time, an untargeted method was tailor-created to annotate, identify and quantify the most abundant compounds among acid triterpenes in *P. heptaphyllum* oleum resin and the two crude extracts by HPLC–HRMS^2^.

A total of 29 molecules were identified and quantified in both resin and extracts, covering the 70% by weight of chemical characterization in *P. heptaphyllum* oleum resin without need for any derivatization protocols for any of the identified molecules. The methods that allowed for the qualitative and qualitative chemical profile obtained in this work represent a solid starting point for the varietal chemical characterization of other Protium species. Furthermore, the application of the developed methods and the use of accurate data on resin composition in future studies will allow a clearer correlation on observed biological activities.

## 6. Patents

Patent related to part of this work is pending, application number 102020000015598.

## Figures and Tables

**Table 1:** Volatile organic compound (VOC) identification and quantification in *Protium heptaphyllum* resin (PHR), acidic triterpene concentrated extract (ATCE) and α and β-amyrin concentrated extract (AMCE) samples.

Compound Name	LRI_C_^2^	LRI_L_^2^	Concentration ^1^					
			PHR		ATCE		AMCE	
α-thujene	926	924	0.49	±0.08	0.19	±0.01	0.97	±0.09
α-pinene	932	932	12.64	±1.91	4.33	±0.07	9.13	±0.71
sabinene	972	969	0.74	±0.15	0.17	±0.02	1.60	±0.22
β-pinene	976	974	1.33	±0.14	0.80	±0.01	1.03	±0.10
α-phellandrene	1005	1002	4.21	±0.13	3.39	±0.05	9.78	±0.71
δ-3-carene	1008	1008	7.04	±0.005	7.34	±0.08	12.14	±1.05
*p*-cymene	1023	1020	11.11	±1.33	9.36	±0.02	3.49	±0.31
β-phellandrene	1028	1025	6.73	±1.11	4.72	±0.03	8.87	±0.79
1,8-cineole	1030	1026	0.75	±0.03	1.09	±0.04	0.12	±0.01
α-terpinolene	1088	1086	0.50	±0.02	0.82	±0.03	0.67	±0.01
*trans-* *Verbenol*	1144	1140	1.29	±0.05	1.78	±0.06	0.00	±0.00
terpinen-4-ol	1177	1174	0.57	±0.10	0.84	±0.01	0.23	±0.03
*p*-cymen-8-ol	1184	1179	1.19	±0.02	1.41	±0.01	0.00	±0.00
β-*e*-caryophyllene	1422	1417	2.15	±0.09	4.58	±0.07	0.69	±0.20
**sum**			50.75	±2.60	40.28	±0.44	48.74	±4.21

^1^ Values are expressed as g kg^−1^ of dry weight and are the mean of at least three replicates ± standard deviation. ^2^ Calculated Linear Retention Index (LRI_C_) with a C_7_–C_30_ *n*-alkanes mixture and the LRI reported in literature (LRI_L_) [16].

**Table 2:** The table reports the validation parameters for amyrin quantitative analyses.

Parameter	α-Amyrin			β-Amyrin			Acceptability Range
*R* ^2^	0.9965 (0.9966)	0.9977 (0.9990)	0.9966 (0.9994)	0.9994 (0.9995)	0.9963 (0.9990)	0.9961 (0.9995)	≥0.995
DIFF%	11.55 (7.15)	3.33 (6.49)	14.89 (13.63)	5.67 (1.52)	10.06 (13.63)	15.73 (15.16)	≤25
SEL%	7.01			7.80			≤30
LOD (mg/L)	0.20 (0.11)			0.24 (0.07)			
LOQ (mg/L)	0.67 (0.39)			0.79 (0.25)			
RSD% 0.5 mg/L (0.1 mg L^−1^)	18.87 (16.95)			16.81 (21.94)			≤25
ERR% 0.5 mg L^−1^ (0.1 mg L^−1^)	13.62 (18.3)	4.66 (25.6)	14.95 (24.6)	13 (12.4)	19.10 (18.4)	8.38 (15.9)	≤20

The reported values are *R*^2^ for each curve, the linearity and repeatability (DIFF%), selectivity (SEL%), sensitivity (limit of detection (LOD), limit of quantitation (LOQ)), percent standard deviation (RSD%) and precision (ERR%) for the unweighted calibration method; values reported in brackets are relative to 1/Y weighted calibration. Parameters are calculated as reported in supplementary materials.

**Table 3:** Quantitation of identified neutral triterpenes in the samples.

Compound	RT (min)	Quantitative Ion (*m/z)*	Concentration (g kg^−1^) ± SD ^1^		
			PHR (*n* = 3)	ATCE (*n* = 4)	AMCE (*n* = 3)
α-amyrin	15.77	218	251.28 ± 19.34	123.98 ±13.92	556.82 ± 30.49
β-amyrin	15.42	218	172.66 ± 21.42	95.39 ± 11.66	385.58 ± 21.82
α-amyron	15.59	218	18.41 ± 0.86	21.71 ± 0.81	n.d. ^2^
β-amyron	15.28	218	14.31 ± 0.61	12.92 ± 6.18	n.d. ^2^
Maniladiol	17.53	234	12.20 ± 1.18	9.36 ± 0.68	17.00 ± 0.90
Brein	18.04	234	14.92 ± 2.27	12.87 ± 0.82	20.88 ± 0.67
Sum (g kg^−1^)			483.77 ± 43.23	270.68 ± 22.75	980.28 ± 51.39

^1^ Quantitative values are expressed as g kg^−1^ and are the mean of, at least, three replicates (*n*) ± standard deviation. ^2^ n.d.: not detected.

**Table 4:** Acidic triterpene features annotation and quantitation.

ID#	Putative Name	Formula	MSI Level	Rt (min)	*m*/*z* [M-H]^−^	MS/MS *(m/z)*	Concentration (g kg^−1^) ± SD					
							PHR *n* = 3	sd	ATCE *n* = 3	sd	AMCE *n* = 3	sd
1	3-oxo -tirucalla-8,24-dien-21-oic acid	C_30_H_46_O_3_	1	15.1	453.3384	435.3234, 371.2563 339.2671	80.64	±3.23	157.10	±0.99	15.31	±0.38
2,3	Oleanolic-Ursolic acid	C_30_H_48_O_3_	1	12.3	455.3510	407.3306	1.85	±0.52	2.40	±0.13	1.93	±0.03
4	3α-Hydroxy-tirucalla-7,24-dien-21-oic acid	C_30_H_48_O_3_	1	14.3	455.3511	437.3391, 373.2720, 339.2676	7.62	±0.18	3.77	±1.21	1.26	±0.45
5	3α-Hydroxy-tirucalla-8,24-dien-21-oic acid	C_30_H_48_O_3_	1	14.9	455.3541	437.3394, 373.2720, 373.2728	77.71	±1.29	130.40	±13.21	11.64	±4.24
6	3β-Hydroxy-tirucalla-8,24-dien-21-oic acid	C_30_H_48_O_3_	1	15.9	455.3540	437.3391, 409.3411, 373.2720	60.14	±9.66	107.87	±7.13	8.38	±2.37
7	Gypsogenin	C_30_H_46_O_4_	2	10.4	469.3324	451.3125, 391.2664, 358.2769	0.34	±0.02	0.35	±0.03	n.d.	n.d.
8	Siaresinol	C_30_H_48_O_4_	2	13.7	471.3490	453.3341, 389.2664, 357.2771	2.99	±0.15	3.73	±0.43	n.d.	n.d.
9	Maslinic Acid	C_30_H_48_O_4_	2	14.6	471.3488	453.3344, 389.2660, 357.2771	2.90	±0.07	4.07	±0.58	n.d.	n.d.
10	3α Acetyl -tirucalla-8,24-dien-21-oic acid	C_32_H_50_O_4_	2	17.1	497.3641	437.3434, 479.3541, 415.2862	4.01	±1.25	6.29	±0.66	n.d.	n.d.
11	3β-Acetyl-tirucalla-8,24-dien-21-oic acid	C_32_H_50_O_4_	2	18.0	497.3640	437.3431, 479.3544, 415.2862	6.62	±2.47	12.71	±2.03	n.d.	n.d.
						Sum (g kg^−1^)	234.65	±14.39	415.26	±18.98	30.23	±6.06

The table reports the identification number (ID#), molecular formula, MSI identification level, retention time (RT), the accurate *m*/*z* and the main fragments of identified compounds; Quantitative values are expressed as g kg^−1^ and are the mean of at least three replicates. ± standard deviation.

**Table 5:** Sample list and description.

Sample Name	Batch	Description
PHR	A0000151	*Protium heptaphyllum* (Aubl.) Marchand oleum resin raw material
ATCE	P75-2-0	Acidic Triterpenes Concentrated Extract obtained from PHR sample
AMCE	P75-2-1	A- and β-Amyrins Concentrated Extract obtained from PHR sample

## Data Availability

The data presented in this study are available on request from the corresponding author. The data are not publicly available because a patent related to a part of this work is pending (application number 102020000015598).

## Supplementary Materials

The following are available online, Table S1: Calibration curves and relative recalculation errors title, Table S2: Weighted calibration curves recalculation errors, Figure S1: Calculating validation parameters, Figure S2: EI–MS signals and proposed fragmentation patterns for (**a**) α-amyrin and (**b**) β-amyrin in samples, molecular ion: 426.44 *m*/*z*, Figure S3: Molecular structures of main identified not-acidic triterpenes in PHR, ATCE and AMCE: (**1**) α-amyrin, (**2**) α-amyron, (**3**) β-amyrin, (**4**) β-amyron, (**5**) Brein and (**6**) Maniladiol.

## Sample Availability

Not available.
