# Supplementary material for: Quali–Quantitative Characterization of Volatile and Non-Volatile Compounds in Protium heptaphyllum (Aubl.) Marchand Resin by GC–MS Validated Method, GC–FID and HPLC–HRMS2
Source: Molecules. 2021 Mar 7;26(5):1447. doi: 10.3390/molecules26051447 (PMC7962098; doi:10.3390/molecules26051447)
Supplement: Supplementary file 1 [file molecules-26-01447-s001.pdf]

Table S1. Unweighted Calibration curves recalculation errors.

	**1st Calibration curve**		**2nd Calibration curve**		**3rd Calibration curve**	
**STD (mg/L)**	A-AMY ERR %	B-AMY ERR %	A-AMY ERR %	B-AMY ERR %	A-AMY ERR %	B-AMY ERR %
**0.1**	130.8*	119.38*	153.03*	130.44*	25.21	49.46*
**0.2**	47.79*	46.67*	63.76*	60.83*	-33.94*	29.80*
**0.5**	-13.62	-13.00	4.66	19.10	-14.95	-8.38
**1**	7.37	10.93	-18.46*	-18.97*	9.20	1.90
**2**	-1.89	-1.92	7.51	-5.30	5.15	6.26
**6**	-11.82	-14.39	0.10	-3.19	1.23	-5.63
**8**	5.63	6.23	1.53	-2.77	8.36	3.94
**10**	-0.04	-0.22	1.27	2.98	-5.81	-0.94

* excluded point.

Table 2. Weighted Calibration curves recalculation errors.

	**1st Calibration curve**		**2nd Calibration curve**		**3rd Calibration curve**	
**STD (mg/L)**	A-AMY ERR %	B-AMY ERR %	A-AMY ERR %	B-AMY ERR %	A-AMY ERR %	B-AMY ERR %
**0.1**	18.3	12.4	25.6	18.4	24.6	-15.9
**0.2**	-5.7	-3.5	-12.7	-1.0	-12.5	5.1
**0.5**	-20.0*	-25.4 *	-17.0*	-7.9	-13.7	16.9*
**1**	-5.7	-2.2	-27.8*	-25.7 *	-3.2	-3.8
**2**	-12.6	-11.2	-10.5	-9.3	5.3	-0.3
**6**	-19.5*	-19.6*	2.6	-1.4	2.3	0.2
**8**	6.9	6.2	-0.4	-1.4	5.9	3.1
**10**	-1.6	-1.9	2.1	4.6	-5.2	-2.6%

* excluded point.

Calculating validation parameters

For a general

$$DIFF\% = \left|\frac{m_i - m}{\underline{m}}\right| \times 100$$

Where:

m_i is the ith calibration line's slope

$\underline{m}$ is the average slope value.

$$LOD = \frac{3\sigma}{m} \qquad LOQ = \frac{10\sigma}{m}$$

Where:

m is calibration line's slope

σ is data's *standard deviation obtained by line equation, more precisely*

$$\sigma = \sqrt{\frac{\sum_{i=1}^{n} \quad (y-y')^2}{n-2}}$$

$$SEL\% = \left|\frac{Peak\ area\ in\ blank\ sample}{3\ peak\ areas\ at\ LOQ}\right| \times 100$$

$$E_r\% = \left|\frac{Theoretical\ analyte\ concentration - True\ analyte\ concentration}{True\ analyte\ concentration}\right| \times 100$$

Er% is obtained for LOQ concentrations

$$RSD\ \% = \frac{\sigma \times 100}{Peaks\ area\ average\ at\ LOQ\ in\ three\ spiked\ samples}$$

Final parameter kept in consideration was recovery; as calculated by internal STD (17-α-methyltestosterone) signal in a STD solution and matrix.

$$REC\ (\%) = \left[\frac{S1}{S2}\right] \times 100$$

Where:

S1 is internal standard signal in matrix

S2 is internal standard signal in standard solution

Figure S1. Formulas for validation parameters.

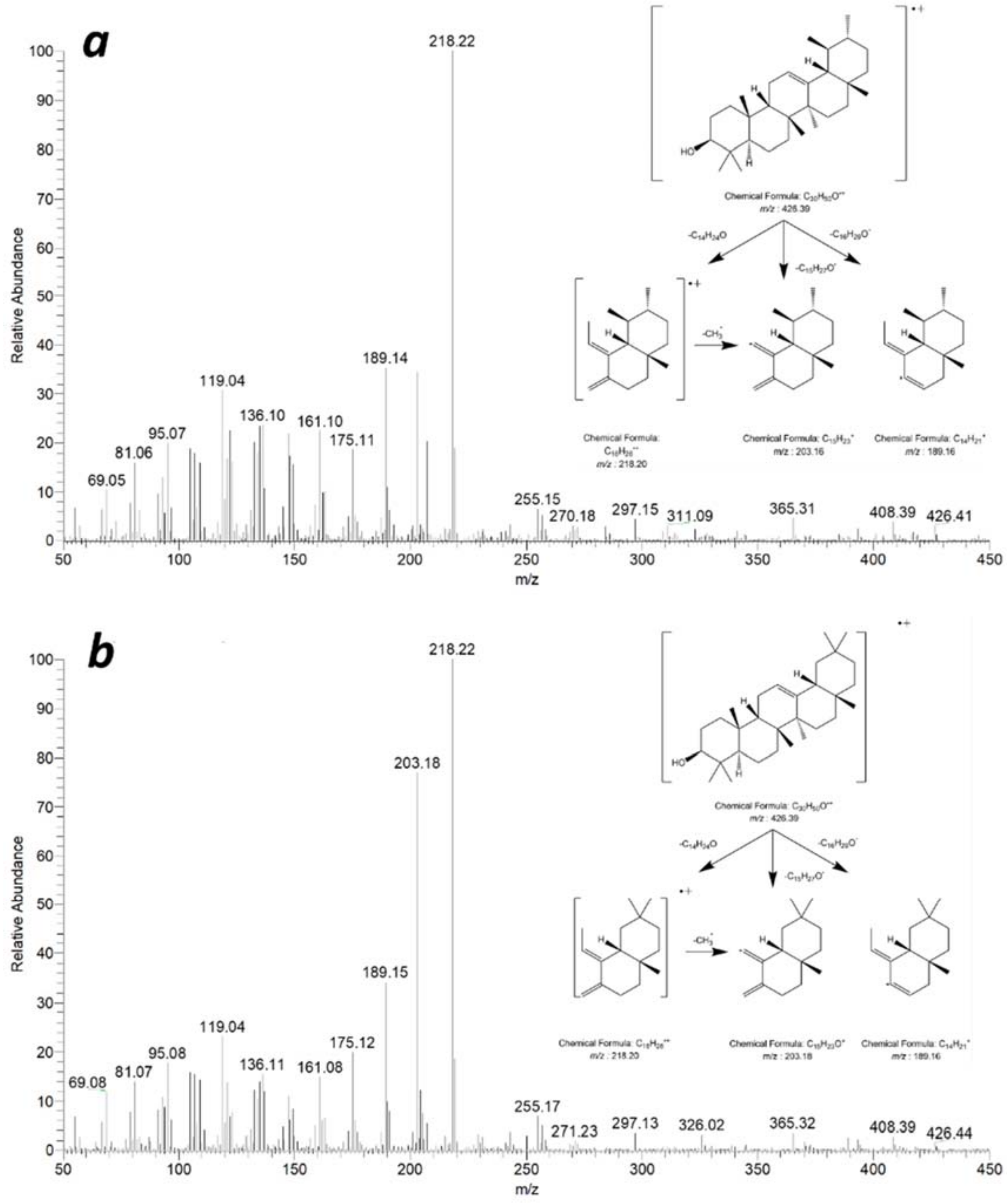

Figure S2. EI-MS signals and proposed fragmentation patterns for (a) *α*-amyrin and (b) β-amyrin in samples, molecular ion: 426.44 *m*/*z*.

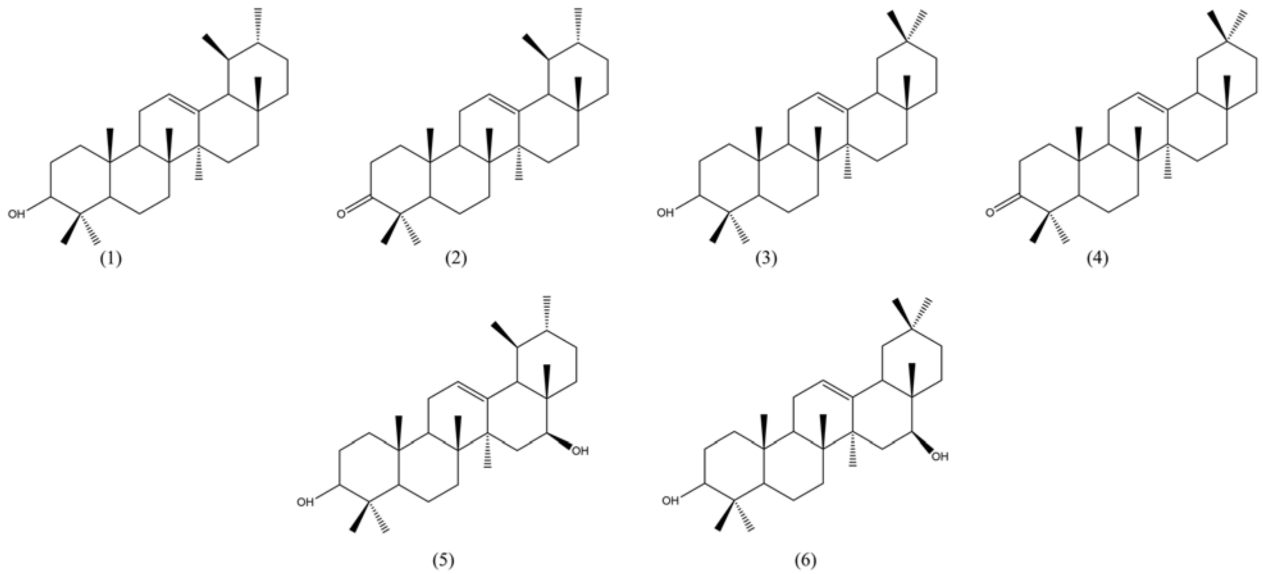

Figure S3. Molecular structures of main identified not acidic triterpenes in PHR, ATCE and AMCE: (1) α-amyrin; (2) α-amyron; (3) β-amyrin; (4) β-amyron; (5) Brein; (6) Maniladiol
